# Wirkungen der Beteiligung und Partizipation von Bürger:innen in Erkenntnisprozessen der integrierten kommunalen Gesundheitsförderung. Ein systematischer Scoping-Review

**DOI:** 10.1007/s00103-025-04013-9

**Published:** 2025-02-18

**Authors:** Susanne Hartung, Stefanie Houwaart, Ursula von Rüden, Ina Schaefer

**Affiliations:** 1 Fachbereich Gesundheit Pflege Management, Hochschule Neubrandenburg, Brodaerstr. 2, 17033 Neubrandenburg, Deutschland; 2 partieval – Vermittlung partizipativer Kompetenzen, Prozessbegleitung und Evaluation im Bereich Gesundheit GmbH, Aachen, Deutschland; 3 Bundeszentrale für gesundheitliche Aufklärung, Köln, Deutschland; 4 Alice Salomon Hochschule Berlin, Berlin, Deutschland

**Keywords:** Gesundheit, Kommune, Partizipative Gesundheitsforschung, Prävention, Setting, Community-based participatory research, Health, Participatory health research, Prevention, Setting

## Abstract

**Hintergrund:**

Beteiligung und Partizipation von Bürger:innen sind anerkannte Anforderungen in verschiedenen Handlungsfeldern von Public Health. Mittels eines Scoping-Reviews sollen die Wirkungen von Beteiligung und Partizipation von Bürger:innen in Erkenntnisprozessen der integrierten kommunalen Gesundheitsförderung systematisch untersucht und aufbereitet werden.

**Methoden:**

Der anhand des PRISMA-Frameworks durchgeführte Scoping-Review wurde durch eine systematische Handrecherche ergänzt. Basierend auf dem Modell der Partizipativen Gesundheitsforschung und dem PHINEO-Wirkungsmodell wurde für die Aufbereitung der Ergebnisse ein Analyseraster entwickelt.

**Ergebnisse:**

Insgesamt wurden 30 Publikationen aus 6 Teilprojekten von Forschungsverbünden (darunter auch forschungsverbundübergreifende Publikationen) sowie aus 6 eigenständigen Projekten in die Auswertung eingeschlossen. Wirkungen wurden hauptsächlich für positive Veränderungen hinsichtlich des Bewusstseins und der Fähigkeiten der direkt beteiligten und partizipierenden Bürger:innen und Projektpartner:innen berichtet sowie darüber hinaus in geringerem Umfang für nicht direkt beteiligte Bürger:innen und Stakeholder. Verhältnisänderungen leiteten sich aus den Veränderungen im Bewusstsein und Handeln der Stakeholder ab, die in den Projekten beteiligt waren.

**Diskussion:**

Die Wirkungen von Beteiligung und Partizipation von Bürger:innen in Erkenntnisprozessen der kommunalen Gesundheitsförderung sind vielfältig und betreffen nicht nur die Zielgruppe des jeweiligen (Teil‑)Projektes. Zugleich gibt es nur wenige Wirkungsbeschreibungen, die über die Ebene der direkt Beteiligten hinausreichen. Für die systematische Implementierung, Evaluation und Veröffentlichung partizipativer Erkenntnisprozesse sind adäquate Förderung und Strukturen nötig.

## Abstract (en)

**Background:**

Participation and its preliminary stages are recognized requirements in various fields of public health. By means of a scoping review, the impact of participation and its preliminary stages of residents in knowledge-generating processes in integrated municipal health promotion are systematically investigated and presented.

**Methods:**

The scoping review was conducted in accordance with the PRISMA framework and extended by systematic manual research. An analysis grid was developed based on the impact model of community-based participatory research and the PHINEO impact model.

**Results:**

In total, 30 publications from six subprojects of research alliances (also publications across research consortia) and six independent projects were analysed. Impact was mostly shown regarding positive changes in awareness and skills for the residents and stakeholder that were directly involved. To a smaller extent, impact was also reported for residents and stakeholders that did not participate in the projects. Changes in conditions and life circumstance for residents resulted from changes in the awareness and actions of the stakeholders involved in the projects.

**Discussion:**

The impact of participation and its preliminary stages of residents in knowledge-generating processes of integrated municipal health promotion is broad and exceeds the target group of the (sub)project. At the same time, impact going beyond the directly involved residents or stakeholders is rarely described. Adequate funding and resources are necessary to systematically facilitate, evaluate and disseminate participatory knowledge-generating processes.

## Hintergrund

Partizipation „ist ein Grundprinzip in allen Public-Health-Handlungsfeldern“, heißt es im Eckpunktepapier der Public-Health-Strategie für Deutschland [1]. Dabei wird betont, dass es einer Stärkung partizipativer Ansätze in den verschiedenen Handlungsfeldern von Public Health bedarf. In der Gesundheitsförderung sind Partizipation und Beteiligung anerkannte Voraussetzungen und etablierte Anforderungen für gelingende Prozesse. Programmatisch verankert ist Partizipation im Sinne der Selbstbestimmung der Bürger:innen als Kern der Gesundheitsförderung [2]. Auch im Präventionsgesetz ist die Aufforderung zur Beteiligung von Versicherten in allen Phasen der Gesundheitsförderung und Prävention in Lebenswelten aufgenommen. In der soziallagenbezogenen Gesundheitsförderung ist Partizipation, verstanden als Entscheidungsteilhabe, bereits lange ein wichtiges Kriterium guter Praxis [3]. Die Argumente für Partizipation sind vielseitig und reichen von direkten gesundheitlichen Wirkungen über die Erhöhung der Bedürfnisgerechtigkeit und Passgenauigkeit bis zur Förderung von gesellschaftlicher Teilhabe und Stärkung der Demokratie [4].

Partizipation kann auf verschiedenen Stufen stattfinden, wobei in der Gesundheitsförderung und partizipativen Forschung erst ab den Stufen von Mitbestimmung, geteilter Entscheidungskompetenz und Entscheidungsmacht von „Partizipation“ gesprochen wird (vgl. Stufenmodell von Wright, von Unger und Block [5, 6]). Dagegen handelt es sich bei Einbeziehung, Anhörung und Information um Vorstufen der Partizipation, die im Weiteren als „Beteiligung“ bezeichnet werden.

Partizipation ist neben verhaltens- und verhältnisbezogenen Maßnahmen und der kontinuierlichen Koordinierung der gesundheitsförderlichen Settingentwicklung eines der Elemente des Settingansatzes [7]. Als Settings werden Sozialzusammenhänge oder Orte bezeichnet, in denen Menschen sich in ihrem Alltag aufhalten und die Einflüsse auf ihre Gesundheit haben. Als Kernstrategie der Gesundheitsförderung wird der Settingansatz insbesondere im sog. Dachsetting Kommune empfohlen. Für die Wirkung von Partizipation auf Zielgruppen und Multiplikator:innen in der Gestaltung von Gesundheitsförderung in einzelnen Settings wie Kitas und Schulen liegen bereits Belege vor [8, 9]. Besondere Wirksamkeit versprechen der Aufbau und die Arbeit entlang integrierter kommunaler Strategien der Gesundheitsförderung (IKS; auch als „Präventionsketten“ bezeichnet; [10–12]). Unter IKS wird „ein gesamtstrategisches Vorgehen zu einer systematischen, ressort- und arbeitsfeldübergreifenden Vernetzung von Gremien, freien Trägern, öffentlichen Institutionen und Zivilgesellschaft durch wirkungsvolle Verbindung und Abstimmung [verstanden. Dabei soll] ein Veränderungsprozess im kommunalen Unterstützungssystem angestoßen [werden], bei dem sowohl die Zusammenarbeit der relevanten Akteurinnen und Akteure als auch konkrete Angebote gemeinsam bedarfs- und bedürfnisgerecht weiterentwickelt“ [13] und die Zielgruppen beteiligt werden [14]. Bei IKS handelt es sich um komplexe und langfristige Interventionen, zu deren Beurteilung der Wirksamkeit und Generierung von Evidenz ein umfassendes und aufwendiges Evaluationsdesign benötigt wird [15].

Die Partizipative Gesundheitsforschung (PGF) untersucht u. a. die Wirkung von Partizipation in der Gesundheitsförderung sowie die förderlichen und hinderlichen Bedingungen für die Partizipation von Zielgruppen, die Formate und Ansprache für Beteiligung [16]. 2007 gründete sich im deutschsprachigen Raum das „Netzwerk Partizipative Gesundheitsforschung“ (PartNet). Forschung und Wissensgenerierung der PGF wird von PartNet „nicht als Privileg von Wissenschaftler:innen, sondern als eine Koproduktion aller beteiligten Forschenden angesehen, die verschiedene Wissens- und Erfahrungsbestände einbringen und zusammenführen. Vor allem die Menschen, deren Leben und Arbeiten unmittelbar von Inhalten und Ergebnissen der Forschung betroffen sind, sollen Einfluss auf den Forschungsprozess nehmen. Die partizipative Forschung hat zwei Zielsetzungen: neue Erkenntnisse zu gewinnen *und* dabei positive Veränderungen anzustoßen. Der Ansatz der Partizipativen Gesundheitsforschung (…) legt den Schwerpunkt auf die Gewinnung von Erkenntnissen, die zur Förderung von Gesundheit und Wohlbefinden der Menschen beitragen und positive Veränderungen, speziell für die Gesundheit sozial benachteiligter Gruppen, anstoßen können“ [17, S. 2, 18, 19]. In Reviews aus dem internationalen Raum wurden bereits vielfältige Wirkungen von PGF u. a. auch im weiteren Kontext von Gesundheitsförderung und Prävention erfasst [20, 21].

Gesundheitsförderung und PGF ist gemeinsam, dass insbesondere die Gruppen beteiligt werden und partizipieren sollen, die von gesundheitlicher Ungleichheit betroffen sind und die im Sinne der PGF an partizipativen Erkenntnisprozessen als Expert:innen mit Erfahrung teilnehmen [22]. Sie werden im Weiteren als Bürger:innen bezeichnet. Dabei können die Bürger:innen zu einer Zielgruppe von Maßnahmen der Gesundheitsförderung gehören, dies muss jedoch nicht notwendigerweise der Fall sein. Auch die Gruppe der Schlüsselakteur:innen, Multiplikator:innen bzw. Stakeholder sollte in die Gestaltung der Gesundheitsförderung durch forschungsbezogene Prozesse in die Phasen der Planung, Umsetzung und Evaluation einbezogen werden. Wenn die Entscheidungsteilhabe bereits bei der Wahl des zu gestaltenden, zu beforschenden Themas und des Forschungs- bzw. Gestaltungsdesigns beginnt, ist von einem größeren Einfluss auf den Erkenntnis- und Gestaltungsprozess auszugehen.

Der vorliegende Scoping-Review, der im Jahr 2023 von der Bundeszentrale für gesundheitliche Aufklärung (BZgA) gefördert wurde, geht der Frage nach: „Welche Wirkungen wurden durch die Beteiligung und Partizipation von Bürgerinnen und Bürgern in Erkenntnisprozessen der integrierten kommunalen Gesundheitsförderung beschrieben?“

Als Erkenntnisprozesse werden Forschungsprozesse der PGF sowie Prozesse aus der Praxis der Gesundheitsförderung verstanden, die einen forschenden Charakter haben, z. B. Bedarfe erheben, Zugangswege zu Zielgruppen analysieren oder Interventionen entwickeln. Mit dieser Begriffserweiterung sollten sowohl Beispiele der PGF als auch der forschenden Praxis der Gesundheitsförderung einbezogen werden, auch wenn sich diese selbst nicht explizit der PGF zuordnen. Wirkungen werden im Scoping-Review als die in den Texten beschriebenen und durch eine Bandbreite von empirischen – meist qualitativen – Methoden dokumentierten Veränderungen verstanden und nicht nur als kausale Wirksamkeitsnachweise [23, 24].

## Methoden

Die Forschungssynthese basiert auf einem Scoping-Review nach Munn et al. [23] und Arksey et al. [24]. Basis sind eine systematische Literaturrecherche in den elektronischen Datenbanken PubMed (Medline), PsycINFO, LIVIVO und Web of Science sowie eine Handrecherche auf Webseiten von Organisationen, wie sie im Folgenden aufgeführt sind:

- Forschungsverbünde zur Präventionsforschung und Forschungsprojekte mit Schwerpunkt Partizipation/kommunale Gesundheitsförderung sowie Universitäten mit Studienangebot im Bereich Public Health/Gesundheitswissenschaften,
- Organisationen auf Landes- und Bundesebene, d. h. Landesvereinigungen für Gesundheit(sförderung) in den Bundesländern und Koordinierungsstellen Gesundheitliche Chancengleichheit, Fonds Gesundes Österreich, Gesundheitsförderung Schweiz,
- Stiftungen mit Förderschwerpunkt kommunale Gesundheitsförderung (z. B. Bertelsmann, Auridis, Radix Schweiz),
- einschlägige Organisationen (u. a. Netzwerk Partizipative Gesundheitsforschung (PartNet), Citizen-Science-Plattform, Netzwerk Caring Communities Schweiz).

Die Ein- und Ausschlusskriterien sind in der Tab. 1 nachzuvollziehen. Ziel war es, alle Projekte im Feld der integrierten kommunalen Strategien der Gesundheitsförderung, die sich der PGF zuordnen lassen und zu denen Veröffentlichungen vorliegen, im Scoping-Review zu erfassen.

**Tab. 1:** Ein- und Ausschlusskriterien für den Scoping-Review

Ein- und Ausschlusskriterien	
Setting	Kommune als Dachsetting, Glieder der „integrierten kommunalen Strategien der Gesundheitsförderung“ (IKS), auch Einzelsettings mit kommunalem Veränderungsbezug, wenn mindestens 2 Personengruppen (Bürger:innen, Praxis und/oder Verwaltung) im Projekt zusammenkamen; nicht eingeschlossen wurden z. B. klinische Studien, epidemiologische Untersuchungen, städteplanerische Maßnahmen ohne Bezug zur Gesundheitsförderung
Studienpopulation	Alle beteiligten und partizipierenden Menschen über alle Lebensphasen hinweg
Studientyp	Peer-geprüfte Publikationen (Einzelstudien, Reviews), Internetquellen („graue Literatur“) und Praxisreports
Zeitraum	Ab dem Jahr 2000
Sprache	Deutsch und Englisch
Geografischer Raum	Deutschsprachiger Raum
Outcomes	Wirkungen der Beteiligung und Partizipation entsprechend Operationalisierung der Fragestellung
Partizipation	Entsprechend der Stufenleiter [5]: Einschluss von Beteiligung (als Vorstufen der Partizipation) und Partizipation; Ausschluss von Nichtpartizipation (z. B. Teilnahme an Studien/Interventionen)

**Abb. 1:**
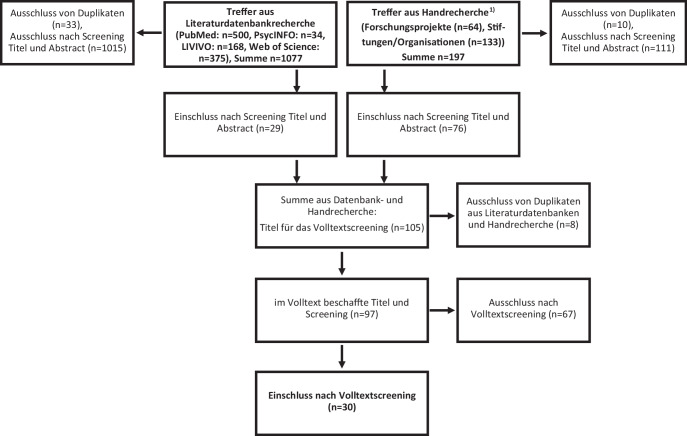
PRISMA-Flussdiagramm [33] der Publikationsauswahl; ^(1)^ als jeweils 1 Treffer wurde die Sichtung von Webseiten gezählt, für die dann keine infrage kommenden Titel recherchiert werden konnten (eigene Abbildung)

Abb. 1 zeigt das PRISMA-Flussdiagramm mit den Auswahlschritten der Publikationen. Von den 1077 Treffern der Literaturdatenbanken wurden 29 Publikationen dem Volltextscreening zugeordnet. Die Qualitätssicherung erfolgte im 4‑Augen-Prinzip für die ersten 265 Treffer [25]. Ein Großteil der Publikationen wurde ausgeschlossen, da sich die in Titel und Abstract beschriebene Partizipation (bzw. engl. „participation“) lediglich auf die Teilnahme an Studien oder Interventionen bezog und weder Beteiligung noch Partizipation umfasste. 105 Titel aus der Literaturdatenbank- und Handrecherche wurden in das Volltextscreening einbezogen. In diesem Schritt wurden 8 Duplikate ausgeschlossen und durch die Analyse der Volltexte anhand der Ausschlusskriterien letztlich 30 Treffer in den Review einbezogen. Die Handrecherche erwies sich als besonders ergiebig. Während in den Literaturdatenbanken wenige Wirkungsbeschreibungen partizipativer Erkenntnisprozesse gefunden werden konnten, stammen 18 der 30 analysierten Volltexte aus der Handrecherche.

**Abb. 2:**
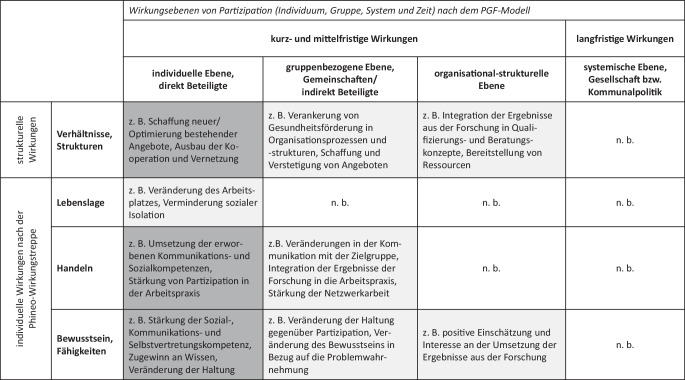
Analyseraster mit Ergebnisüberblick zu Wirkungen von Beteiligung und Partizipation in Erkenntnisprozessen kommunaler Gesundheitsförderung, entwickelt von Hartung, Houwaart und Schaefer (eigene Abbildung). *Weiß* unterlegte Felder mit *n.* *b.* keine Veränderungen berichtet; *hellgrau *unterlegte Felder: Veränderungen in weniger als der Hälfte der analysierten Forschungsverbünde und Projekte berichtet; *dunkelgrau* unterlegte Felder: Veränderungen in der Hälfte oder mehr der analysierten Forschungsverbünde und Projekte berichtet; *PGF* Partizipative Gesundheitsforschung

Die 30 Volltexte wurden mithilfe der qualitativen Inhaltsanalyse [26] ausgewertet. Für die Extraktion der Ergebnisse war es notwendig, die Darstellungen in den analysierten Publikationen konzeptionell in Bezug auf Partizipation und integrierte kommunale Strategien der Gesundheitsförderung zu verorten. Außerdem wurden die möglichen Wirkungen von Partizipation in ein Analyseraster gebracht, welches dann auch zur Darstellung der Ergebnisse diente (Abb. 2).

Für die Entwicklung dieses Rasters wurde erstens das Modell für Partizipative Gesundheitsforschung (PGF-Modell; [27, 28]) und zweitens die Differenzierung individueller Wirkungen aus der Wirkungstreppe von Phineo [29] herangezogen. Aus dem PGF-Modell wurden die Differenzierung zwischen kurz-/mittelfristigen und langfristigen Wirkungen (systemische Ebene der Gesellschaft und Politik, z. B. Veränderungen in der Kommunalpolitik eines Landkreises) sowie die Differenzierung zwischen Wirkungen auf verschiedene Beteiligtengruppen (Tab. 2) aufgegriffen. Als Beteiligtengruppen wurden direkt und indirekt beteiligte Bürger:innen sowie direkt und indirekt beteiligte Stakeholder unterschieden. Als Stakeholder wurden die Praktiker:innen in kommunalen Einrichtungen sowie in der kommunalen Verwaltung bezeichnet. Diese Fachkräfte beeinflussen das Gelingen partizipativer Prozesse und sind die entscheidenden Akteur:innen, um Veränderungen in der sozialen Lebenswelt zu bewirken. Als indirekt Beteiligte wurden diejenigen angesehen, die nicht selbst an den partizipativen (Forschungs‑)Prozessen beteiligt waren, an die aber die partizipativ erarbeiteten Ergebnisse gerichtet waren, um Veränderungen zu bewirken, beispielsweise die betreffenden Einrichtungen eines Trägers in der Kommune. Auch bei der organisational-strukturellen Ebene handelt es sich um indirekt Beteiligte, wenn z. B. Veränderungen trägerübergreifend in den betreffenden Einrichtungen einer Kommune angestoßen werden. Insofern hat diese Ebene eine größere Reichweite.

**Tab. 2:** Übersicht über Ebenen der kurz-/mittelfristigen Wirkungen auf unterschiedliche Personengruppen

Ebenen der kurz-/mittelfristigen Wirkungen	Personen/-gruppen
Individuelle Ebene der direkt Beteiligten	Bürger:innen: Zielgruppenvertreter:innen und/oder Expert:innen aus Erfahrung
	Stakeholder: Praktiker:innen in Einrichtungen der Kommune, Akteur:innen der kommunalen Verwaltung
Gruppenbezogene Ebene, Gemeinschaften der indirekt Beteiligten	Bürger:innengruppe(n)
	Akteur:innen in der betreffenden kommunalen Verwaltung
Organisational-strukturelle Ebene	Vertretungen der Bürger:innen
	Einrichtungen in der Kommune
	Kommunale Verwaltung

Wirkungen wurden nach der Wirkungstreppe von Phineo [29] als Veränderungen im Bewusstsein und den Fähigkeiten, im Handeln und in der Lebenslage differenziert. Diese individuellen Wirkungsdimensionen wurden zusätzlich durch die Dimension der Verhältnisse und Strukturen ergänzt, um gemäß dem Settingansatz auch Wirkungen in den Lebens- und Arbeitsbedingungen als strukturelle Faktoren aufnehmen zu können.

Für die mithilfe der Software MAXQDA durchgeführte Codierung der analysierten Volltexte wurden die Codes mehrheitlich deduktiv aus der Forschungsfrage, den im Analyseraster dargestellten Ebenen der Wirkungen sowie möglichen Veränderungen abgeleitet. Einzelne weitere induktive Codes wurden während des Codiervorgangs nach gemeinsamer Absprache im Projektteam hinzugenommen. Testung, Abstimmung und Anpassung der Codes wurden von 2 Personen vorgenommen.

Für die Auswertung und differenzierte Darstellung der Wirkungen waren die Überschneidungen der Codes für die einzelnen Ebenen (z. B. kurzfristige Wirkungen, individuelle Ebene und direkt Beteiligte z. B. „Praktiker:in in kommunaler Einrichtung“) und die unterschiedlichen Veränderungen (z. B. „Veränderung im Handeln“) von besonderer Bedeutung. Diese Überschneidungen wurden mithilfe der komplexen Segmentsuche in MAXQDA identifiziert und ausgelesen. Alle weiteren für die Beantwortung der Forschungsfrage und die Darstellung der Studienexzerpte relevanten Textpassagen wurden anhand ihrer spezifischen Codes ausgelesen und für die Ergebnisdarstellung kategorisiert.

Die Codierung der Texte erfolgte nach der Abstimmung anteilig durch 2 Personen. Die jeweiligen Codes wurden zunächst tabellarisch zusammengefasst. Daraus wurde in extrahierter Form eine beschreibende Zusammenfassung der Veränderungen auf den verschiedenen Ebenen entwickelt. Zusätzlich wurden die recherchierten Projekte auch einzeln zusammenfassend beschrieben.

## Ergebnisse

### Überblick über die identifizierten Projekte

Die Ergebnisse des Scoping-Reviews werden als Überblick über die identifizierten Projekte und zugehörigen 30 Volltexte in Tab. 3 präsentiert. Auffällig war, dass ein Großteil der Publikationen aus Forschungsverbünden stammt, die zwischen 2014 und 2023 vom Bundesministerium für Bildung und Forschung (BMBF) in der Förderlinie der Primärprävention und Gesundheitsförderung finanziert wurden. Von diesen gingen Publikationen von 6 Teilprojekten aus den Forschungsverbünden AEQUIPA, Capital4Health und PartKommPlus in die Auswertung ein. Einbezogen wurden außerdem forschungsverbundübergreifende Publikationen von Capitel4Health und der Koordinierungsstelle von PartKommPlus. Dazu umfasst die Volltextanalyse 6 Einzelprojekte. Alle einbezogenen Projekte sind als Forschungsprojekte zu verstehen, die Interventionen im Feld der kommunalen Gesundheitsförderung entwickeln, umsetzen und/oder evaluieren. In den Projekten wurden überwiegend sowohl verhaltens- als auch verhältnispräventive Maßnahmen in den Blick genommen und die Aktivitäten gingen in der Regel über die Grenzen eines einzelnen Settings hinaus. Die verhaltenspräventiven Maßnahmen bezogen sich in diesen Projekten auf verschiedene Handlungsfelder, vor allem auf Bewegungsförderung und Stärkung von Gesundheitskompetenz, des Weiteren auf gesundheitsförderliche Ernährung, Stressreduzierung und Entspannung sowie Suchtprävention. In Tab. 3 sind der Fokus der Maßnahmen und der jeweilige Settingbezug aufgeführt. Mit der Angabe „Unterstützung bei Aufbau und Stärkung gesundheitsförderlicher Strukturen“ werden auch Ansätze bezeichnet, die soziale bzw. umweltbezogene Gesundheitsdeterminanten adressieren.

**Tab. 3:** Übersicht über die einbezogenen Volltexte des Scoping-Reviews, die Projekte, primäre Zielgruppen, Beteiligung und Partizipation sowie Basis der Wirkungsbeschreibung^a^

Projekte (bzw. forschungsverbundübergreifende Betrachtungen)	Publikationen	Fokus der Maßnahmen nach GKV (2023) und Settingbezügen	Primäre Zielgruppe	Beschreibung der Beteiligung/Partizipation	Basis der Beschreibung von Wirkungen der Partizipation
				(1) Beteiligung/Partizipation Phase Planung, Umsetzung und/oder Evaluation	Angabe der Methode zur Wirkungsmessung und/oder Darstellung der Wirkung als Reflexionsbeschreibungen in den Publikationen, wenn keine spezifischen Angaben zu der/den Methode/n der Wirkungsbeschreibung verfügbar
				(2) Beteiligung/Partizipation Themensetzung und/oder Design	
*Teilprojekte aus Forschungsverbund PartKommPlus*					
Koordinierung PartKommPlus	PartKommPlus (2021; [33]) Brandes et al. (2021; [34])	– Unterstützung bei Aufbau und Stärkung gesundheitsförderlicher Strukturen – Kommunale Gesundheitsförderung, verschiedene Zielgruppen	– Wissenschaft – Stakeholder – Bürger:innen	(1) (2)	– Wirkungskarten (Impact-Mapping) – Analyse verbundinterner Dokumente – Reflexionsbeschreibungen
Eltern fragen Eltern (ELFE)	Bär et al. (2021; [9]) Bär et al. (2021; [35]) Bär et al. (2021; [36]) Harms (2019; [37]) Hilgenböcker et al. (2021; [38])	– Unterstützung bei Aufbau und Stärkung gesundheitsförderlicher Strukturen – Kommune, Quartier – Kita	– Eltern mit Kindern im Kita-Alter	(1) (2)	u. a.: – Leitfadeninterviews – Gruppendiskussion – Dokumentenanalyse – Impact-Gespräche
Menschen mit Lernschwierigkeiten und Gesundheitsförderung (GESUND!)	Becker/Burtscher (2019; [39]) Burtscher (2019; [40]) Allweiss et al. (2021; [41])	– Angebote Bewegungsförderung – Angebote gesundheitsförderliche Ernährung – Angebote Stressreduktion und Ressourcenstärkung – Stärkung der Gesundheitskompetenz – Unterstützung bei Aufbau und Stärkung gesundheitsförderlicher Strukturen – Kommune, Quartier – Werkstatt für Menschen mit Behinderung	– Menschen mit Lernschwierigkeiten	(1) (2)	– Partizipative Schreibwerkstätten – Wirkungskarte – Dokumentenanalyse – Standardisierte Fachkräftebefragung
Kommunale Entwicklung von Gesundheitsstrategien: Wissenschaft und Praxis im Dialog (KEG)	PartKommPlus (2023; [42]) Wihofszky et al. (2021; [43])	– Unterstützung bei Aufbau und Stärkung gesundheitsförderlicher Strukturen – Kommune, Stadtteil	– Jugendliche – Jugendliche in sozial und gesundheitlich belasteten Lebenslagen	(1) (2)	– Regelmäßige Reflexionen – Reflexionsbeschreibungen
Partizipative Evaluation der Präventionskette Braunschweig (PEPBS)	PartKommPlus (2021; [44]) Rataj et al. (2021; [45]) Wihofszky et al. (2020; [46])	– Unterstützung bei Aufbau und Stärkung gesundheitsförderlicher Strukturen Kommune – Schule (Praxisklasse) – Offene Jugendtreffs	– Jugendliche – Jugendliche in sozial und gesundheitlich belasteten Lebenslagen	(1)	– Standardisierte Befragung (Online) – Leitfadeninterviews – Reflexionsworkshops
*Teilprojekte aus Forschungsverbund Capital4Health*					
Forschungsverbundübergreifend	Gelius et al. (2021; [47]) Gelius et al. (2021; [48]) Sauter und Loss (2021; [49])	– Angebote Bewegungsförderung – Unterstützung bei Aufbau und Stärkung gesundheitsförderlicher Strukturen – Kommunale Gesundheitsförderung, verschiedene Zielgruppen	– Wissenschaft – Stakeholder – Bürger:innen	(1)	– Dokumentenanalyse – Leitfadeninterviews – Reflexionsbeschreibungen
Action for Men (Action4men)	Loss et al. (2020; [50])	– Angebote Bewegungsförderung – Unterstützung bei Aufbau und Stärkung gesundheitsförderlicher Strukturen – Kommune	– Körperlich inaktive Männer über 50 Jahre	(1)	– Teilstandardisierter Dokumentationsbogen – Teilstrukturierte Interviews
*Teilprojekte aus Forschungsverbund AEQUIPA*					
Entwicklung eines gemeindebasierten Programms zur Förderung der Outdooraktivität bei älteren Erwachsenen 65+ (OUTDOOR ACTIVE)	Bammann et al. (2021; [51])	– Angebote Bewegungsförderung – Unterstützung bei Aufbau und Stärkung gesundheitsförderlicher Strukturen – Kommune, Bezirk	– Menschen älter als 65 Jahre	(1)	– Reflexionsbeschreibungen
*Einzelne Projekte*					
Bewegung in Vorschule	De Bock et al. (2013; [52])	– Angebote Bewegungsförderung – Kita mit Bezug zur kommunalen Umgebung durch initiierte Projekte	– Kinder im Vorschulalter (4–6 Jahre)	(1)	– Randomisierte kontrollierte Studien (RCT) – Standardisierte Prozessdokumentation durch Auswertung der standardisierten Protokolle basierend auf dem RE-AIM-Ansatz („reach, effectiveness, adoption, implementation and maintenance“)
Bewegung als Investition in Gesundheit (BIG)	Herbert-Maul et al. (2020; [53]) Herbert-Maul et al. (2021; [54]) Rütten et al. (2023; [55]) Sauter et al. (2022; ([56)]) Till et al. (2023; [57])	– Angebote Bewegungsförderung – Unterstützung bei Aufbau und Stärkung gesundheitsförderlicher Strukturen – Kommune, Stadtteil	– Frauen in schwierigen Lebenslagen	(1)	– Teilstandardisierte Interviews – Dokumentenanalysen
Gesunder Schlaf	Landwehr (2022; [58])	– Angebote Stressreduktion und Ressourcenstärkung – Kommune – Schule	– Kinder im Grundschulalter (3. Klasse)	(1)	– Standardisierte Fragebögen – Selbstreflexion
Migrant/innen aus Subsahara-Afrika (MiSSA)	Kuehne et al. (2018; [59])	– Stärkung der Gesundheitskompetenz – Unterstützung bei Aufbau und Stärkung gesundheitsförderlicher Strukturen – Kommune	– Migrant:innen aus Subsahara-Afrika	(1)	– Reflexionsbeschreibungen für die Ebene der Wissenschaft
Partizipation, Suchtprävention und Migration (PaSuMi)	Gangarova/Schäffer (2020; [60])	– Suchtprävention – Stärkung der Gesundheitskompetenz – Unterstützung bei Aufbau und Stärkung gesundheitsförderlicher Strukturen – Kommune – Einrichtungen der Suchtprävention und -hilfe	– Geflüchtete, sexarbeitende, drogenkonsumierende, substituierte und/oder HIV- und/oder HCV-positive Migrant:innen	(1)	u. a. – Standardisierte Fragenbögen – Dokumentenanalysen – Fokusgruppendiskussion
Zämegolaufe	Frei et al. (2019; [61])	– Angebote Bewegungsförderung, Unterstützung bei Aufbau und Stärkung gesundheitsförderlicher Strukturen – Kommune	– Personen über 60 Jahren	(1)	– Reflexionsbeschreibungen

^a^ Im Forschungsprojekt wurden 2 weitere Fragestellungen bearbeitet, die an dieser Stelle nicht dargestellt werden. Durch diese Fragestellungen wurde eine Veröffentlichung in den Scoping-Review aufgenommen, welche die Einschlusskriterien erfüllt, aber nur Wirkungen für die Ebene der Wissenschaft zeigt

Gemäß den Einschlusskriterien wurden nur Publikationen berücksichtigt, die sich auf partizipative Erkenntnisprozesse mit Bürger:innen (Zielgruppen, Expert:innen aus Erfahrung) und/oder Stakeholdern (Praxisakteur:innen in Einrichtungen und der kommunalen Verwaltung) im Feld der kommunalen Gesundheitsförderung beziehen und über einzelne Settings hinausgehende Bezüge aufweisen. Eine Eingrenzung auf Projekte mit direktem Bezug zu integrierten kommunalen Strategien der Gesundheitsförderung war nicht möglich, da IKS lediglich in den Teilprojekten von PartKommPlus direkt adressiert wurden. Als „primäre Zielgruppe“ werden in Tab. 3 die Personengruppen bezeichnet, an die sich das Projekt hauptsächlich richtete.

Partizipation wurde in den verschiedenen Projekten und Phasen der Erkenntnis- und Interventionsprozesse in sehr unterschiedlicher Intensität umgesetzt. Im Detail kann die Umsetzung der Partizipation auf Basis der geprüften Publikationen nicht nachvollzogen werden. In Tab. 3 wird jedoch ausgewiesen, ob Beteiligung und Partizipation in den Phasen Planung, Umsetzung und/oder Evaluation sowie hinsichtlich der Themenwahl und Entscheidungen über das Untersuchungsdesign erfolgte. Die Untersuchung der Wirkungen von Beteiligung und Partizipation wurde in den Projekten überwiegend durch ein qualitatives Vorgehen (z. B. durch Dokumentenanalysen, Fokusgruppendiskussionen und Interviews) umgesetzt, nur in einem Fall in Form einer randomisierten kontrollierten Studie (RCT). Dabei wurden die Wirkungen einer partizipativen Intervention im Vergleich zu einer expertengetriebenen Intervention untersucht [30]. Zudem wurden Wirkungen aufgenommen, die z. B. basierend auf einer Reflexion der Erfahrungen im Prozess in den Texten beschrieben wurden. Diese werden in Tab. 3 als „Reflexionsbeschreibungen“ bezeichnet.

Der Fokus lag insgesamt auf Wirkungen, die dem partizipativen Erkenntnisprozess zugeordnet werden können, auch wenn diese Differenzierung gegenüber den Wirkungen der Intervention nicht immer trennscharf möglich ist. Wirkungen durch die Intervention selbst (z. B. gesteigertes Bewegungsverhalten) werden nur dann berichtet, wenn diese im Vergleich zu nicht partizipativ entwickelten Interventionen dargestellt werden.

### Wirkungen der Beteiligung und Partizipation

Abb. 2 stellt das zuvor beschriebene Raster für die Analyse der Volltexte sowie einen Ergebnisüberblick dar. Die Markierung der Felder über die Farbgebung verweist auf die Häufigkeit, in der in den analysierten Projekten und Forschungsverbünden Wirkungen auf den verschiedenen Ebenen und für die unterschiedlichen Beteiligtengruppen berichtet wurden. Ein Rückschluss auf die Quantität der beschriebenen Wirkungen und ebenso auf die Qualität des methodischen Vorgehens für die Wirkungserhebung ist nicht möglich. Beispielsweise kann es sich bei beschriebenen Veränderungen in der Lebenslage der direkt Beteiligten auch um aus subjektiver Sicht beschriebene Wirkungen auf einzelne Personen handeln. Die weißen Tabellenfelder mit der Abkürzung „n. b.“ (nicht berichtet) zeigen, dass auf diesen Ebenen jeweils keine Veränderungen in den analysierten Volltexten beschrieben werden. In den hellgrau unterlegten Feldern wurden in weniger als der Hälfte, in den dunkelgrau unterlegten Feldern in der Hälfte bzw. mehr als der Hälfte der analysierten Projekte und Forschungsverbünde Veränderungen berichtet. Außerdem werden in den hell- und dunkelgrau unterlegten Feldern jeweils Beispiele angegeben, welche Wirkungen hier berichtet wurden.

Während nahezu alle recherchierten Projekte Veränderungen auf Ebene der direkt Beteiligten (beteiligte Bürger:innen und/oder Praktiker:innen in Einrichtungen und/oder Akteur:innen der kommunalen Verwaltung) beschreiben, sind es deutlich weniger Projekte, die Veränderungen auf Ebene der nicht direkt Beteiligten sowie auf der organisational-strukturellen Ebene berichten. Auf der langfristigen systemischen Ebene wurden keine Wirkungen berichtet.

Die überwiegende Mehrheit der auf individueller Ebene der direkt Beteiligten berichteten Wirkungen sind Veränderungen in Bewusstsein und Fähigkeiten. Die beschriebenen Veränderungen im Handeln der Bürger:innen bezogen sich größtenteils auf die Umsetzung der durch Beteiligung und Partizipation neu hinzugewonnenen Kompetenzen. Eine Änderung der Lebenslage der beteiligten Bürger:innen wird nur in einzelnen Projekten und für einzelne Personen beschrieben (z. B. Zugang zum Arbeitsmarkt).

Ebenfalls beschreiben mehr als die Hälfte der analysierten Projekte strukturelle Wirkungen auf Ebene der direkt Beteiligten, beispielsweise in Form neuer Angebote. Die Veränderungen in den Verhältnissen und Strukturen waren oft Konsequenzen aus den Veränderungen im Bewusstsein und dem Handeln der Praktiker:innen oder der Personen, die in den kommunalen Verwaltungen arbeiten und in den Projekten partizipierten oder mit ihnen im Austausch standen. Entwickelten diese Gruppen ein Verständnis für die Perspektive der Bürger:innen und nahmen sie diese mit ihren Herausforderungen, aber auch Kompetenzen wahr, so konnte Vertrauen aufgebaut werden. Das wiederum resultierte in neuen Beziehungskonstellationen, z. B. zwischen Fachkräften und Jugendlichen. Die durch die Zusammenarbeit entstandene Vernetzung, z. B. zwischen den Bürger:innenvertretungen und kommunaler Politik, führte zu mehr Mitsprache der Bürger:innen in kommunalen Angelegenheiten, konkret in den einzelnen kommunalen Settings. Auch die durch partizipative Prozesse angestoßene Entwicklung neuer kommunaler Angebote stellte für die Verhältnisse der Bürger:innen eine positive Veränderung dar.

## Diskussion

Im Rahmen des Scoping-Reviews wurden 3 Forschungsverbünde mit 6 Teilprojekten sowie 6 weitere Forschungsprojekte identifiziert, in denen mit Bezug zur kommunalen Gesundheitsförderung partizipativ mit Bürger:innen als Zielgruppe der Intervention zusammengearbeitet wurde und in denen die Wirkungen der Beteiligung und Partizipation beschrieben wurden.

Obgleich partizipative Erkenntnisprozesse mit Wirkungsbeschreibungen, die vonseiten der Praxis initiiert und durchgeführt wurden, ausdrücklich Gegenstand des Scoping-Reviews sein sollten, konnten entsprechende Publikationen weder in den Literaturdatenbanken noch im Rahmen der umfassenden Handrecherche identifiziert werden. Hinzu kommt, dass auch Einzelsettings mit kommunalem Veränderungsbezug in die Recherche aufgenommen wurden, da sich nur wenige partizipative Projekte zur kommunalen Gesundheitsförderung explizit in IKS verorten. Der Scoping-Review bildet daher nur partizipative Forschungsprojekte in verschiedenen Handlungsfeldern der Gesundheitsförderung und Prävention ab, die im kommunalen Setting verortet sind und damit einzelne Glieder der Präventionskette als IKS darstellen können. Mit dem Ergebnis der Literaturrecherche kann die Frage, wie Beteiligung und Partizipation in IKS umgesetzt werden, nicht direkt beantwortet werden, es lassen sich aber Anhaltspunkte aus dem Scoping-Review finden.

Inwieweit die berichteten Wirkungen auf Beteiligung und Partizipation oder auf die gesundheitsförderliche Intervention zurückzuführen sind, lässt sich nicht präzise differenzieren. Die Ergebnisse internationaler Reviews [20, 21] zeigen vergleichbare Wirkungen partizipativer Forschungsprozesse. Insofern kann geschlussfolgert werden, dass die berichteten Wirkungen auf Partizipation und partizipative Forschungsprozesse zurückzuführen sind und nicht allein auf gesundheitsförderliche Intervention. Die Wirkungen von Intervention und Partizipation getrennt zu erfassen, bedürfte eines in der Praxis hochkomplexen und kaum realisierbaren experimentellen Verfahrens.

Partizipation, zumal im Rahmen von kommunaler Gesundheitsförderung, ist ressourcenaufwendig. Nicht verwunderlich ist es daher, dass die berichteten Wirkungen ausschließlich aus Forschungsprojekten stammen. Dies zeigt, dass Forschung zu diesen Fragen weiterhin notwendig und sinnvoll ist. Wirkung lässt sich in der Praxis nicht nebenbei erheben und publizieren. Hierfür sind spezifische Ressourcen erforderlich.

Es wurden wertvolle Hinweise generiert, welche Wirkungen durch Partizipation in der kommunalen Gesundheitsförderung erwartet werden können. Die Wirkungen im Bewusstsein und den Fähigkeiten der direkt Beteiligten sind am einfachsten zu erheben. Methodisch deutlich aufwendiger ist das Generieren von Evidenz hinsichtlich der Veränderungen im Handeln. Gleiches gilt für die langfristigen Wirkungen auf systemischer Ebene. Dabei zeigen einzelne Projekte, dass zumindest mittelfristige Wirkungen auf organisational-struktureller Ebene geplant und erzielt werden können, wenn die kommunale Verwaltung als Stakeholder in die Projekte einbezogen wird.

Sowohl für die Praxis der kommunalen Gesundheitsförderung als auch für die hier angesiedelte Forschung ist es eine Herausforderung, sich in multiprofessionellen Teams mit Bürger:innen und Stakeholdern mit den Zielen und beabsichtigten Wirkungen auseinanderzusetzen. Dies zeigt sich in der partizipativ vs. explizit nichtpartizipativ arbeitenden Praxis der kommunalen Gesundheitsförderung. Die Arbeit mit Wirkmodellen – und hier auch die Einbeziehung von Beteiligung und Partizipation – kann bereits in der Projektplanung eine systematisch auf die Erreichung von Wirkungen gerichtete Arbeitsweise fördern. In der fortlaufenden Evaluation kann sie zu einer besseren Darstellung auch der durch Beteiligung und Partizipation erzielten Veränderungen beitragen. Die Forschung kann die Praxis hierbei umfassender unterstützen, wenn diese Begleitung frühzeitig und langfristig mitgedacht und finanziert wird. Beispielhaft konnte dies bereits an der Förderung der Präventionskette Niedersachsen gezeigt werden [10].

Unser Review zeigt auch, dass die adressat:innengerechte Verbreitung der Erkenntnisse für Veränderungen notwendig ist. Die Analyse der Volltexte ergab, dass eine Wirkung auf Ebene der indirekt Beteiligten dann berichtet wurde, wenn in dem Projekt auch eine Disseminationsphase angelegt war. Allerdings wird bzw. kann die spezifische und gezielte Dissemination von Projektergebnissen bei kurzen und mittleren Projektlaufzeiten nur selten in Angriff genommen werden. Erst dadurch aber wird eine über das Projekt hinausgehende Evaluation von Wirkungen sinnvoll (auch ohne die genuin Projektbeteiligten). Entsprechend sind längerfristige Projektanlagen erforderlich, um gezielter Wirkungen auf den verschiedenen Ebenen anstreben, realisieren und evaluieren zu können.

### Abgeleitete Handlungsempfehlungen

Aus den Ergebnissen lassen sich Handlungsempfehlungen ableiten. So sollten die für Forschungs- und Erkenntnisprozesse im Bereich der kommunalen Gesundheitsförderung zur Verfügung gestellten Ressourcen auch an den Spezifika des partizipativen Ansatzes ausgerichtet werden, indem ermöglicht wird, dass

- auch Bürger:innen und Stakeholder bereits in die Entwicklung von Forschungsanträgen (und damit auch der übergeordneten Zielsetzung) eingebunden werden, sodass deren Perspektive von Beginn an Berücksichtigung finden kann,
- dem erhöhten Moderations- und Ermöglichungsaufwand (Facilitating, Kapazitätsaufbau) partizipativer Prozesse Rechnung getragen wird, sodass auch eine umfassende Auseinandersetzung mit der Wirkungslogik stattfinden kann,
- eine Unterstützung des partizipativen Erkenntnisprozesses durch Expert:innen für Wirkungsorientierung und Evaluation partizipativer Prozesse gegeben ist,
- die adressatenspezifische Dissemination explizit gefördert wird, u. a. mit Ressourcen für die Erarbeitung der Ansprache und des Layouts,
- in Forschung investiert wird, die die Konzeption von Wirkungen in partizipativen Erkenntnisprozessen weiterentwickelt und auch Indikatoren für die Evaluation künftiger Wirkungsüberprüfungen ableitet.

Es wird außerdem empfohlen, die Forschung zu den Langzeiteffekten von Partizipation in Erkenntnisprozessen der kommunalen Gesundheitsförderung, zur diesbezüglichen Nachhaltigkeit und zum breiteren sozialen Impact der Beteiligung von Bürger:innen an IKS zu fördern. Für die Umsetzung von Partizipation in IKS wurden im Review Anhaltspunkte gefunden. Die Erforschung von Langzeiteffekten der Partizipation kann dazu beitragen, die Argumente für IKS weiter zu stärken.

Wie für die kommunale Ebene ist auch für die Forschungsförderung durch nationale Akteur:innen eine verbesserte Sichtbarkeit von Wirkungsbeschreibungen aus der Praxis – im Sinne von praxisbasierter Evidenz [31] – in der Wissenschaftscommunity zu ermöglichen sowie in einen besseren Zugang der Praxis zu wissenschaftlichen Publikationen zu investieren. Damit verknüpft sind die Publikation in wissenschaftlichen Open-Access-Formaten sowie die Förderung von Publikationen in Formaten, die in der Praxis verbreitet sind und von Praktiker:innen gelesen werden.

### Limitationen

In den analysierten Publikationen wurde überwiegend nicht transparent genug dargestellt, welches Partizipationsverständnis zugrunde gelegt und wie Partizipation genau umgesetzt wurde. Zwar war in der von uns durchgeführten Analyse eine diesbezügliche Rückbindung an die jeweiligen Konzepte angelegt. Doch auch mit einem höheren zeitlichen Aufwand konnte dies aufgrund der fehlenden Information nicht vollständig umgesetzt werden. Auch adressierten die Projekte in der Regel nicht explizit das Konzept der IKS und z. T. auch nicht der kommunalen Gesundheitsförderung. Für künftige Darstellungen der Wirkungen von Beteiligung und Partizipation von Bürger:innen an Erkenntnisprozessen der (integrierten) kommunalen Gesundheitsförderung ist es wünschenswert, dass die Verortung in den verschiedenen Konzepten transparenter gemacht wird und auch über deren Umsetzung, insbesondere der Partizipation, berichtet wird.

Die Evaluation der kurz- und mittelfristigen und besonders der langfristigen Wirkungen durch partizipative Erkenntnisprozesse auf den verschiedenen Ebenen (integrierter) kommunaler Gesundheitsförderung ist in der Regel nicht Gegenstand der Projektförderung, was die Trefferquote nachvollziehbar einschränkte. Eine Herausforderung bei der Erfassung von Wirkungen durch partizipative Forschung besteht im Weiteren darin, dass Kausalitäten zwischen den Forschungsprozessen und ihren Wirkungen schwer nachweisbar sind und Wirkungen selten eindeutig zugeordnet werden können [32]. Hinzu kommt, dass der Aufwand für die Evaluation je nach Ebene, die in den Blick genommen wird, ansteigt. Eine Evaluation der Wirkungen auf die direkt Beteiligten lässt sich mit weniger Aufwand in ein Projekt integrieren als beispielsweise die Evaluation der Wirkungen auf die indirekt Beteiligten. Aus den Ergebnissen sollte daher nicht geschlussfolgert werden, dass Veränderungen, über die nicht berichtet wird, nicht stattgefunden haben. Möglich ist auch, dass sie lediglich nicht evaluiert wurden. Ohne die Mindestvoraussetzung der gezielten Veröffentlichung und Dissemination der gewonnenen Erkenntnisse in die spezifische Lebenswelt kann jedoch keine Wirkung erwartet werden.

## Fazit

Das für den Scoping-Review entwickelte Modell möglicher Veränderungen durch partizipative Erkenntnisprozesse im kommunalen Setting basiert auf dem Wirkungsverständnis der Gesundheitsförderung sowie dem Wirkungsmodell der Partizipativen Gesundheitsforschung und bietet eine gute Grundlage, um Wirkungen der partizipativen, kommunalen Gesundheitsförderung auf individueller, gruppenbezogener und struktureller Ebene zu operationalisieren.

Zugleich ist sowohl die Umsetzung der Wirkungsorientierung als auch der Partizipation sehr voraussetzungsreich. In den IKS wird zwar ein Schwerpunkt auf die Wirkungsorientierung gelegt [8]. Allerdings ist die explizite Verortung von IKS in einem partizipativen Ansatz eher selten, auch wenn Partizipation als Kriterium in den einzelnen Maßnahmen in der Regel mitgedacht ist.

Partizipative Arbeitsweisen stellen zeitlich wie finanziell hohe Anforderungen an alle Beteiligten. Die Ergebnisse des Reviews stärken aber die Argumentation, dass sich dieser Aufwand lohnt. Partizipative Prozesse der Planung, Umsetzung und Evaluation können eigene positive Wirkungen entfalten und Wirkungen von Interventionen in Handlungsbereichen der Gesundheitsförderung verstärken.

## References

[1] Zukunftsforum Public Health (2021) Eckpunkte einer Public-Health-Strategie für Deutschland. https://zukunftsforum-public-health.de/download/eckpunkte-einer-public-health-strategie-langversion/. Zugegriffen: 10. Juli 2024

[2] Weltgesundheitsorganisation (1986) Ottawa-Charta zur Gesundheitsförderung. https://iris.who.int/handle/10665/349652. Zugegriffen: 20. Dez. 2023

[3] Kooperationsverbund Gesundheitliche Chancengleichheit (2021) Kriterien für gute Praxis der soziallagenbezogenen Gesundheitsförderung, Kriterium „Partizipation“. https://www.gesundheitliche-chancengleichheit.de/fileadmin/user_upload/pdf/Good_Practice/21-08-30_Broschuere_Good_Practice-Kriterien_neu_barrierefrei_01.pdf. Zugegriffen: 20. Dez. 2023

[4] Rosenbrock R, Hartung S (2012) Handbuch Partizipation und Gesundheit. Huber, Bern

[5] Wright M, von Unger H, Block M (2010) Partizipation der Zielgruppe in der Gesundheitsförderung und Prävention. In: Wright MT (Hrsg) Partizipative Qualitätsentwicklung in der Gesundheitsförderung und Prävention. Huber, Bern, S 35–52

[6] Wright M (2021) Partizipative Gesundheitsforschung: Ursprünge und heutiger Stand. Bundesgesundheitsblatt Gesundheitsforschung Gesundheitsschutz 64:140–145. 10.1007/s00103-020-03264-y 33336312 10.1007/s00103-020-03264-y PMC7843534

[7] Hartung S, Rosenbrock R (2022) Settingansatz-Lebensweltansatz. 10.17623/BZGA:Q4-i106-2.0. Zugegriffen: 10. Juli 2024

[8] Funk S, Schaefer I, Kolip P (2019) Was fördert die Verstetigung von Strukturen und Angeboten der Gesundheitsförderung? Gesundheitswesen. 10.1055/s-0042-116437 27846667 10.1055/s-0042-116437

[9] Bär G, Fiebig P, Katsch K et al (2021) Partizipativ arbeiten für kommunale Strategien der Gesundheitsförderung. Sozialmagazin 11–12:35–42. 10.3262/SM2112035

[10] Brandes S (2023) EvaluationsReport zur Prozessevaluation des Programms „Präventionsketten Niedersachsen: Gesund aufwachsen für alle Kinder!“. Subjektive Wahrnehmung verschiedener Akteur*innen zu Aspekten der Vernetzung. https://www.praeventionsketten-nds.de/unser-programm/evaluationsreport/. Zugegriffen: 20. Dez. 2023

[11] Brandes S (2024) Evaluationsreport: FOKUS Dialoggruppen; Familien im Quartier, Laatzen. https://nbn-resolving.org/urn:nbn:de:0168-ssoar-94146-3. Zugegriffen: 10. Juli 2024

[12] Brandes S (2024) Evaluationsreport: FOKUS Dialoggruppen; „Groß und Klein – keiner allein“, Barsinghausen. https://nbn-resolving.org/urn:nbn:de:0168-ssoar-94147-8. Zugegriffen: 10. Juli 2024

[13] Richter-Kornweitz A, Holz G, Kilian A (2023) Präventionskette – Integrierte kommunale Gesamtstrategie zur Gesundheitsförderung und Prävention. In: Bundeszentrale für gesundheitliche Aufklärung (BZgA) (Hrsg) Leitbegriffe der Gesundheitsförderung und Prävention. Glossar zu Konzepten, Strategien und Methoden 10.17623/BZGA:Q4-i093-2.0

[14] Hartung S (2020) Prävention und Gesundheitsförderung in Kommunen. In: Tiemann M, Mohokum M (Hrsg) Prävention und Gesundheitsförderung. Springer Reference Pflege – Therapie – Gesundheit. Springer, Berlin, Heidelberg, S 1–11

[15] Humrich W, Kilian H, Richter-Kornweitz A et al (2024) Wirkungsorientierung in Gesundheitsförderung und Prävention. Präv Gesundheitsf. 10.17623/BZGA:Q4-i161.10

[16] Hartung S, Wihofszky P, Wright MT (2020) Partizipative Forschung. Ein Forschungsansatz für Gesundheit und seine Methoden. Springer VS, Wiesbaden

[17] Hartung S, Wihofszky P, Wright MT (2020) Partizipative Forschung – ein Forschungsansatz für Gesundheit und seine Methoden. In: Hartung S, Wihofszky P, Wright MT (Hrsg) Partizipative Forschung. Springer VS, Wiesbaden, S 1–19

[18] PartNet – Netzwerk Partizipative Gesundheitsforschung (2015) Partizipative Gesundheitsforschung – eine Definition. http://partnet-gesundheit.de/ueber-uns/partnet-definition/. Zugegriffen: 20. Dez. 2023

[19] Hartung S, Landwehr J, Schaefer I (2025) Erläuterung der PartNet-Definition zur Partizipativen Gesundheitsforschung. Konzeptionelle Grundlagen und spezifische Elemente. PartNet Perspektiven. Beiträge zur partizipativen Forschung 01/25. 10.58123/aliceopen-654

[20] Jagosh J, Macaulay A, Pluye P et al (2012) Uncovering the benefits of participatory research: implications of a realist review for health research and practice. Milbank Quarterly 90:311–346. 10.1111/j.1468-0009.2012.00665.x 22709390 10.1111/j.1468-0009.2012.00665.x PMC3460206

[21] Hoekstra F, Mrklas KJ, Khan M et al (2020) A review of reviews on principles, strategies, outcomes and impacts of research partnerships approaches a first step in synthesising the research partnership literature. Health Res Policy Syst. 10.17605/OSF.IO/GVR7Y 32450919 10.1186/s12961-020-0544-9 PMC7249434

[22] Wahl A, Kasberg A, Netzelmann T et al (2021) PartNet-Diskussionspapier: Beteiligte an Partizipativer Gesundheitsforschung. 10.17883/2434 (PartNet Perspektiven. Beiträge zur partizipativen Forschung 1/21)

[23] Munn Z, Pollock D, Khalil H et al (2022) What are scoping reviews? Providing a formal definition of scoping reviews as a type of evidence synthesis. JBI Evid Synth 20:950–952. 10.11124/jbies-21-00483 35249995 10.11124/JBIES-21-00483

[24] Arksey H, O’Malley L (2005) Scoping studies: towards a methodological framework. Int J Soc Res Methodol 8:19–32. 10.1080/1364557032000119616

[25] Peters M, Godfrey C, McInerney P et al (2020) Scoping reviews. In: Aromataris EMZ (Hrsg) JBI manual for evidence synthesis. JBI, S 406–451

[26] Mayring P (2010) Qualitative Inhaltsanalyse. In: Mey G, Mruck K (Hrsg) Handbuch Qualitative Forschung in der Psychologie. VS, Wiesbaden, S 601–613

[27] Schaefer I, Allweiss T, Dresen A et al (2022) PartNet-Methodenpapier: Modell für Partizipative Gesundheitsforschung (PGF-Modell). 10.17883/2762. (PartNet Perspektiven. Beiträge zur partizipativen Forschung 02/22)

[28] Wallerstein N, Oetzel J, Duran B et al (2008) What predicts outcomes in CBPR? In: Minkler M, Wallerstein N (Hrsg) Community-based participatory research for health. From process to outcomes. Jossey-Bass, San Francisco, Calif, S 371–392

[29] PHINEO (2021) Kursbuch Wirkungen – Das Praxishandbuch für alle, die Gutes noch besser tun wollen. https://www.phineo.org/kursbuch-wirkung. Zugegriffen: 10. Juli 2024

[30] Bock C, Jarczok M, Litaker D (2014) Community-based efforts to promote physical activity: a systematic review of interventions considering mode of delivery, study quality and population subgroups. J Sci Med Sport 17:276–282. 10.1016/j.jsams.2013.04.009 23693030 10.1016/j.jsams.2013.04.009

[31] Hartung S (2021) Praxisbasierte Evidenz in Public Health. In: Schmidt-Semisch H, Schorb F (Hrsg) Public Health. Disziplin – Praxis – Politik. Springer VS, Wiesbaden, S 349–369

[32] Allweiss T, Cook T, Wright MT (2021) Wirkungen in der partizipativen Gesundheitsforschung: Eine Einordnung in die Diskurse zum Forschungsimpact. Bundesgesundheitsblatt Gesundheitsforschung Gesundheitsschutz 64:215–222. 10.1007/s00103-020-03268-8 33373016 10.1007/s00103-020-03268-8 PMC7843532

[33] Ziegler A, Antes G, König IR (2011) Bevorzugte Report Items für systematische Übersichten und Meta-Analysen: Das PRISMA-Statement. Dtsch Med Wochenschr. 10.1055/s-0031-127297821332033

[34] PartKommPlus (2021) Wirkungsbeschreibung. PartKommPlus – Forschungsverbund für gesunde Kommunen. https://www.partkommplus.de/fileadmin/files/Dokumente/Anhaenge_fuer_Wirkungsbeschreibungen/21-02-04_ANHANG_Langfassung_der_Wirkungsbeschreibung_PartKommPlus_01.pdf. Zugegriffen: 20. Dez. 2023

[35] Brandes M, Muellmann S, Allweiss T et al (2021) Evidence-based primary prevention and health promotion: methods and procedures in 5 research consortia. Bundesgesundheitsblatt Gesundheitsforschung Gesundheitsschutz 64(5):581–589. 10.1007/s00103-021-03322-z 33835197 10.1007/s00103-021-03322-z PMC8033542

[36] Bär G, Katsch K, Orschmann S et al (2021) Wirkungsbeschreibung des Teilprojekts „ElfE – Eltern fragen Eltern“. https://www.partkommplus.de/fileadmin/files/Dokumente/ElfE/Impact_Narrativ_ElfE_Langfassung.pdf. Zugegriffen: 20. Dez. 2023

[37] Bär G, Reutlinger C (2021) Manövrieren zwischen gesellschaftlichem Wandel, Lernen und der Generierung neuen Wissens – Das Bermudadreieck der partizipativen Forschung. In: Flick S, Herold A (Hrsg) Zur Kritik der partizipativen Forschung – Forschungspraxis im Spiegel der Kritischen Theorie. Beltz Juventa, Weinheim Basel, S 156–184

[38] Harms R, Bär G (2019) Kompetenzentwicklung durch partizipative Forschung. https://www.partkommplus.de/fileadmin/files/Dokumente/ElfE/Elfe-Infoblaetter/Kompetenzentwicklung_durch_partizipative_Forschung_2019.pdf. Zugegriffen: 20. Dez. 2023

[39] Hilgenböcker E, Bär G, Kühnemund C (2021) Verstetigung partizipativer Forschung über das Projektende hinaus: Partizipative Qualitätsentwicklung in der kommunalen Gesundheitsförderung. Bundesgesundheitsblatt Gesundheitsforschung Gesundheitsschutz 64(2):207–214. 10.1007/s00103-020-03271-z 33399944 10.1007/s00103-020-03271-z

[40] Becker K, Burtscher R (2019) Gemeinsam forschen – Gemeinsam lernen. Menschen mit Lernschwierigkeiten in der Partizipativen Gesundheitsforschung. Stiftung Rehabilitationszentrum Berlin-Ost, Berlin

[41] Burtscher R (2019) Wirkungen und Gelingensbedingungen der Partizipativen Gesundheitsforschung. In: Walther K, Römisch K (Hrsg) Gesundheit inklusive. Springer VS, Wiesbaden, S 89–106

[42] PartKommPlus (2021) Wirkungsbeschreibung des PartKommPlusTeilprojekts „GESUND!“. Projekt GESUND! https://www.partkommplus.de/fileadmin/files/Dokumente/Anhaenge_fuer_Wirkungsbeschreibungen/21-06-02_Wirkungsbeschreibung_GESUND.pdf. Zugegriffen: 21. Dez. 2023

[43] PartKommPlus (2023) Wirkungsbeschreibung Projekt Kommunale Entwicklung von Gesundheitsstrategien: Wissenschaft und Praxis im Dialog (KEG). https://www.partkommplus.de/wirkungen/wirkungsbeschreibungen/keg-wirkungsbeschreibung/index.html. Zugegriffen: 20. Dez. 2023

[44] Wihofszky P, Hofrichter P, Layh S et al (2021) Transfer partizipativer Forschungsergebnisse in die Praxis: Das Beratungsinstrument Standortanalyse in der kommunalen Gesundheitsförderung. Bundesgesundheitsblatt Gesundheitsforschung Gesundheitsschutz 64:199–206. 10.1007/s00103-020-03273-x 33403461 10.1007/s00103-020-03273-x PMC7785035

[45] PartKommPlus (2021) Wirkungsbeschreibung des Teilprojekts „Partizipative Evaluation der Präventionskette Braunschweig“ (PEPBS^2^). https://www.partkommplus.de/wirkungen/wirkungsbeschreibungen/pepbs2-wirkungsbeschreibung/index.html. Zugegriffen: 20. Dez. 2023

[46] Rataj E, Fischer J, Bogner A et al (2021) Autonomie in der Offenen Kinder- und Jugendarbeit fördern – Ergebnisse eines partizipativen Evaluationsprojekts. Bundesgesundheitsblatt Gesundheitsforschung Gesundheitsschutz 64:179–186. 10.1007/s00103-020-03275-9 33415382 10.1007/s00103-020-03275-9 PMC7790478

[47] Wihofszky P, Hartung S, Allweiss T et al (2020) Photovoice als partizipative Methode: Wirkungen auf individueller, gemeinschaftlicher und gesellschaftlicher Ebene. In: Hartung S, Wihofszky P, Wright MT (Hrsg) Partizipative Forschung: Ein Forschungsansatz für Gesundheit und seine Methoden. Springer VS, Wiesbaden, S 85–141

[48] Gelius P, Brandl-Bredenbeck HP, Hassel H et al (2021) Kooperative Planung von Maßnahmen zur Bewegungsförderung. Bundesgesundheitsblatt Gesundheitsforschung Gesundheitsschutz 64:187–198. 10.1007/s00103-020-03263-z 33315164 10.1007/s00103-020-03263-z PMC7843529

[49] Gelius PC, Sommer RM, Abu-Omar K et al (2021) Toward the economic evaluation of participatory approaches in health promotion: lessons from four German physical activity promotion projects. Health Promot Int 36:ii79–ii92. 10.1093/heapro/daab158 34905608 10.1093/heapro/daab158 PMC8670626

[50] Sauter A, Loss J (2021) Capacity building in participatory stakeholder groups: results from a German research consortium on active lifestyles. Health Promot Int 36:65–78. 10.1093/heapro/daab165 10.1093/heapro/daab165 PMC8672937 34905613

[51] Loss J, Brew-Sam N, Metz B et al (2020) Capacity building in community stakeholder groups for increasing physical activity: results of a qualitative study in two German communities. Int J Environ Res Public Health 17:17. 10.3390/ijerph17072306 10.3390/ijerph17072306 PMC7177804 32235419

[52] Bammann K, Recke C, Albrecht B et al (2021) Promoting physical activity among older adults using community-based participatory research with an adapted PRECEDE-PROCEED model approach: the AEQUIPA/OUTDOOR ACTIVE project. Am J Health Promot 35:409–420. 10.1177/0890117120974876 33267636 10.1177/0890117120974876 PMC8010898

[53] De Bock F, Genser B, Raat H et al (2013) A participatory physical activity intervention in preschools: a cluster randomized controlled trial. Am J Prev Med 45:64–74. 10.1016/j.amepre.2013.01.032 23790990 10.1016/j.amepre.2013.01.032

[54] Herbert-Maul A, Abu-Omar K, Frahsa A et al (2020) Transferring a community-based participatory research project to promote physical activity among socially disadvantaged women-experiences from 15 years of BIG. Front Public Health 8:571413. 10.3389/fpubh.2020.571413 33072709 10.3389/fpubh.2020.571413 PMC7542241

[55] Herbert-Maul A, Abu-Omar K, Streber A et al (2021) Scaling up a community-based exercise program for women in difficult life situations in Germany-the BIG project as a case-study. Int J Environ Res Public Health 18:15. 10.3390/ijerph18189432 10.3390/ijerph18189432 PMC8468862 34574356

[56] Rütten A, Semrau J, Wolff A (2024) Entwicklung gesundheitsförderlicher Strukturen durch kooperative Planung. Präv Gesundheitsf 19:233–242. 10.1007/s11553-023-01045-4

[57] Sauter A, Herbert-Maul A, Abu-Omar K et al (2022) „For me, it’s just a piece of freedom“-Increased empowerment through physical activity promotion among socially disadvantaged women. Front Public Health 10:12. 10.3389/fpubh.2022.867626 10.3389/fpubh.2022.867626 PMC9363839 35968425

[58] Till M, Abu-Omar K, Gelius P (2023) Der Capability-Ansatz in der Bewegungsförderung. Präv Gesundheitsf 18:111–118. 10.1007/s11553-022-00934-4

[59] Landwehr J (2022) Die Perspektive von Grundschulkindern auf gesunden Schlaf – eine partizipative Pilotstudie mit Photovoice. Forum Qual Sozialforsch. 10.17169/fqs-23.3.3879

[60] Kuehne A, Koschollek C, Santos-Hövener C et al (2018) Impact of HIV knowledge and stigma on the uptake of HIV testing—results from a community-based participatory research survey among migrants from sub-saharan africa in Germany. PLoS ONE 13:e194244. 10.1371/journal.pone.0194244 29641527 10.1371/journal.pone.0194244 PMC5894987

[61] Gangarova T, Schäffer D (2020) Partizipative und Diversity-orientierte Entwicklung der Suchtprävention und Suchthilfe für und mit Migrant/innen (PaSuMi). https://www.bundesgesundheitsministerium.de/fileadmin/Dateien/5_Publikationen/Drogen_und_Sucht/Berichte/Abschlussbericht/PaSuMi_Abschlussbericht_bf.pdf. Zugegriffen: 20. Dez. 2023

[62] Frei A, Dalla LK, Radtke T et al (2019) A novel approach to increase physical activity in older adults in the community using citizen science: a mixed-methods study. Int J Public Health 64:669–678. 10.1007/s00038-019-01230-3 30937463 10.1007/s00038-019-01230-3

