# Erratum zu: Wirkungen der Beteiligung und Partizipation von Bürger:innen in Erkenntnisprozessen der integrierten kommunalen Gesundheitsförderung. Ein systematischer Scoping-Review

**DOI:** 10.1007/s00103-026-04206-w

**Published:** 2026-03-05

**Authors:** Susanne Hartung, Stefanie Houwaart, Ursula von Rüden, Ina Schaefer

**Affiliations:** 1 Fachbereich Gesundheit Pflege Management, Hochschule Neubrandenburg, Brodaerstr. 2, 17033 Neubrandenburg, Deutschland; 2 partieval – Vermittlung partizipativer Kompetenzen, Prozessbegleitung und Evaluation im Bereich Gesundheit GmbH, Aachen, Deutschland; 3 Bundeszentrale für gesundheitliche Aufklärung, Köln, Deutschland; 4 Alice Salomon Hochschule Berlin, Berlin, Deutschland

**Erratum zu:**

**Bundesgesundheitsbl 2025**

10.1007/s00103-025-04013-9

In Tab. 3 fehlten in der Kopfzeile von Spalte 5 die Ziffern (1) und (2). Außerdem wurden in der Tabelle fälschlicherweise eingefügte Trennlinien wieder entfernt. Der Originalbeitrag wurde korrigiert.

